# A Physician’s Estimate of Modern Dentistry

**Published:** 1916-01

**Authors:** 


### A PHYSICIAN'S ESTIMATE OF MODERN DENTISTRY.

Woods Hutchinson, A.M., M.D., recently discussing the profession of dentistry and the relative susceptibility to dental disease of ancient and modern man, came to the rather unusual conclusion that prehistoric evidence is far from convincing that the teeth of moderns suffer more from the ravages of disease than those of the ancient. Dr. Hutchinson says:

The evidence for the decline of modern teeth which is furnished by an examination of ancient or prehistoric skulls dug up or uncovered in chance excavations is at first sight rather discouraging. The great majority of these are remarkably well stocked with teeth in a very fair state of repair. The percentage of missing or defective teeth is, on the whole, surprisingly small, ranging from 15 or 20 per cent. to as low as two per cent. And dental experts who have examined large collections of these skulls declare that hollow teeth or signs of dental abscess are less than half as common as they would be in a similar body of adults in a dental clinic today. But the first thing that strikes us about these ancient skulls is that the overwhelming majority of them are of men, and of men in the prime of young adult life at that—very few women's skulls, and practically no children's skulls at all. This used to be explained on the ground that they were from soldiers killed in some great battle, even though no record or legend had survived of a battle at that spot. But so constant is this overwhelming preponderance of young male skulls in all large collections dug up in the open earth that we are beginning to strongly suspect that we are dealing with a survival of only the strongest and solidest skulls, which would naturally be those of young men. And as the foundation and solidest part of the skull is its jaws, and the jaws depend entirely upon the teeth and waste away when the teeth are lost, the skull which would have the best chance of surviving would be, first of all, the young male adult; second, the young male adult skull which had the best and most perfect set of teeth.

At all events we are entitled to the consolation of knowing that even in this probably highly selected class of skulls, the overwhelming majority of which in any case are adult males in the prime of life, those who have survived the perils of childhood and adolescence and have not yet been decayed by the degeneration of advancing years—even among this group of "champion" skulls, there are to be found every type of dental defect, or abscess, of pulp abscess, of indications showing that the teeth were lost by pyorrhea, of malpositions, and irregularities of the teeth, and of failures of the jaws to grip and and grind firmly and evenly one upon the other—technically known as malocclusion—which are known to civilized dentistry. So the difference between ancient and modern teeth shipwreck at best is only one of degree, not of kind.

But, when we lament that things were never half so bad as this in our father's or our grandfather's days, we are going beyond our evidence, because no examinations were ever made then. The teeth of school children only began to be systematically examined about fifteen or twenty years ago, and until about twenty-five or thirty years ago, no recruiting surgeon ever looked at a volunteer's teeth, except just to see that he had enough front teeth to tear open his old-fashioned paper cartridge with. Incidentally, it may be remarked that the great importance now attached to the condition of the teeth in recruits accounts for nine-tenths of the difference between the large number of rejections today and the smaller number fifty years ago.

### MODERN METHODS FOR SCHOOL CHILDREN.

Thanks to the magnificent work now being done in school dental clinics and mouth hygiene movements, so that in some progressive towns and districts all the children's teeth in the community are being systematically and thoroughly examined, treated and recorded, we shall, twenty years from now have the data to make a comparison which will really give us reliable information. Personally, I don't mind hazarding the prophecy that it will show distinct improvement instead of deterioration.

THE PRACTICE OF MODERN DENTISTRY.

It must be remembered that it is only about thirty-five or forty-five years ago that the science of modern dentistry was born, and that dentists were invented and began to go about with a holy zeal for improvement, and tell us what terrible sets of teeth we kept. And few things have added more already to the comfort, the efficiency and the good looks of mankind than that same invention and crusade. Indeed, it is not too much to say that modern science has already checked or begun to check whatever decadence of modern teeth may have set in. How this has been done is almost a household word. Not only has the school dentist and the army dentist and the prison dentist become a regular and indispensable institution, but many of our more intelligent employers of labor on a larger scale are establishing dental rooms and dental clinics upon their premises. Among the latest to join the procession are department stores, life insurance company headquarters and manufacturers. Fully equipped dental rooms are provided in the building, with dentists in constant attendance, who examine the teeth of all employes and do prophylactic or preventive work and then either refer cases requiring more extensive treatment to their private dentist, or make arrangements to do the work at moderate rates and let payments be made in weekly installments.

Applicants for employment, if found satisfactory in other respects, are sent to the dentist for examination, and if dental defects are found, they are required to have these corrected, or to agree to have them attended to within a reasonable time, before they are appointed to a position. The rèasons for this precaution and expenditure are straightforward and practical ones: First, that it has been found that putting the teeth in good condition and keeping them so improves the health and working power of the employee, and distinctly diminishes the number of days of absence on account of sickness throughout the year. The same had already been found true in school children in several of our cities, those who had gone to the dentist and had their teeth attended to, showing less than half the number of absences of those who had failed to heed such directions.

GOOD TEETH A BUSINESS ASSET.

The other reason is equally practical, though an esthetic one, and that is the much more attractive appearance presented by employes with white, lustrous and even sets of teeth, and the fact that customers prefer to be waited upon by clerks whose teeth are clean and fresh and wholesome looking, as well as their complexion and finger nails and attire. Besides, the girl who knows she has pretty teeth is usually more willing to display them in a smile, and as she is also free from the carping discomforts of toothache and ulcerated gums she is more likely to be affable and obliging in her demeanor.

In fact, these higher standards of dental deportment, both individual and general, have produced an appreciable effect already, and nowhere in the world can one see so universally perfect and sparkling and attractive sets of teeth as upon the principal shopping streets of Canadian and American cities. And one of the most universal comments made on our women and girls by the visiting foreigners is the beauty, whiteness and evenness of their teeth.—*Oral Health.*

